# Intermittent fasting prompted recovery from dextran sulfate sodium-induced colitis in mice

**DOI:** 10.3164/jcbn.17-9

**Published:** 2017-07-28

**Authors:** Toshihiko Okada, Takeshi Otsubo, Teruki Hagiwara, Fumika Inazuka, Eiko Kobayashi, Shinji Fukuda, Takuya Inoue, Kazuhide Higuchi, Yuki I. Kawamura, Taeko Dohi

**Affiliations:** 1 Department of Gastroenterology, Research Center for Hepatitis and Immunology, Research Institute, National Center for Global Health and Medicine, 1-7-1 Kohnodai, Ichikawa, Chiba 282-8516, Japan; 2 2nd Department of Internal Medicine, Osaka Medical College, 2-7 Daigakumachi, Takatsuki, Osaka 569-8686, Japan; 3 Institute for Advanced Biosciences, Keio University, 246-2 Mizukami, Kakuganji, Tsuruoka, Yamagata 997-0052, Japan

**Keywords:** lactate, interleukin-1β, interleukin-17, NDRG3, colitis

## Abstract

Fasting-refeeding in mice induces transient hyperproliferation of colonic epithelial cells, which is dependent on the lactate produced as a metabolite of commensal bacteria. We attempted to manipulate colonic epithelial cell turnover with intermittent fasting to prompt recovery from acute colitis. Acute colitis was induced in C57BL/6 mice by administration of dextran sulfate sodium in the drinking water for 5 days. From day 6, mice were fasted for 36 h and refed normal bait, glucose powder, or lactylated high-amylose starch. On day 9, colon tissues were subjected to analysis of histology and cytokine expression. The effect of lactate on the proliferation of colonocytes was assessed by enema *in vivo* and primary culture *in vitro*. Intermittent fasting resulted in restored colonic crypts and less expression of interleukin-1β and interleukin-17 in the colon than in mice fed *ad libitum*. Administration of lactate in the colon at refeeding time by enema or by feeding lactylated high-amylose starch increased the number of regenerating crypts. Addition of lactate but not butyrate or acetate supported colony formation of colonocytes *in vitro*. In conclusion, intermittent fasting in the resolution phase of acute colitis resulted in better recovery of epithelial cells and reduced inflammation.

## Introduction

Accumulating evidence points to the emerging importance of dietary interventions, including intermittent fasting, calorie restriction, and protein restriction, in various inflammatory diseases.^(1,2)^ For example, in rats and mice with experimental allergic encephalomyelitis (EAE), calorie restriction prevented the disease through reduced production of interferon (IFN)-γ.^(3,4)^ In a mouse EAE model, intermittent feeding with access to food on alternate days significantly reduced the incidence of EAE,^(5)^ and a ketogenic diet improved motor disability.^(6)^ Recent reports indicated that fasting mimicking diet cycles lowered visceral fat, reduced cancer incidence and skin lesions, rejuvenated the immune system, and retarded bone mineral density loss.^(7)^ Furthermore, fasting has been reported to ameliorate demyelination and symptoms of mouse EAE, associated with increased corticosterone levels and regulatory T (Treg) cell numbers and reduced levels of pro-inflammatory cytokines, TH1 and TH17 cells, and antigen-presenting cells.^(8)^ In humans, accumulated evidence suggests that fasting reduces both inflammation and pain in patients with rheumatoid arthritis and that it might be useful in their treatment.^(9)^ In an infectious colitis model, calorie restriction decreased the survival rate in SMAD3−/− mice infected with *Helicobacter hepaticus*.^(10)^ In dextran sulfate sodium (DSS)-induced colitis in mice, a high-protein diet during the induction and resolution periods increased inflammation intensity compared with a normoproteinic diet, but it resulted in higher colonic epithelial repair in surviving animals.^(11)^ The mammalian target of rapamycin (mTOR) signaling pathway, a sensor of nutritional condition and regulator of cell metabolism, is now known to control immune and inflammatory responses.^(12)^ It was reported that treatment of mouse DSS colitis with an mTOR inhibitor, which blocks nutritional signals, ameliorated inflammation with decreased leukocyte migration^(13)^ or colonic expression of IFN-γ, interleukin (IL)-17A, IL-1β, IL-6 and tumor necrosis factor (TNF)-α, and increased IL-10 in the colon.^(14)^

We previously found that a fasting-refeeding cycle induced transient arrest of epithelial cell (EC) division and then hyperproliferation of colonic ECs in a mouse model.^(15)^ This fluctuation included two distinct phases of colonic EC turnover: 1) total arrest of cell division during fasting and 2) hyperproliferation 12–24 h after refeeding with three-fold increase in the number of mitotic cells compared with *ad libitum*-fed individuals. This variation strikingly influences the outcome of exposure to mutagens. For example, repeated injection of the carcinogen azoxymethane in phase 2 doubled the number of precancerous lesions in the colon compared with *ad libitum*-fed mice. On the other hand, fasting-refeeding after exposure to azoxymethane almost completely suppressed the development of precancerous lesions.^(15)^ Fasting also protected the intestine of the mice from gamma-ray irradiation or DNA damage by etoposide during fasting (our unpublished data and Ref^(16)^). This cycle of cell turnover rate was produced by the synchronized alteration of colonic microbiota to eating behavior, and we showed that lactate, produced by intestinal microbiota Lactobacillus [*Lactobacillus murinus* (*L. murinus*)] with metabolizing dietary fiber, induced colonic EC hyperproliferation *in vivo*. Treatment of mice with oral antibiotics eliminated the hyperproliferation in the refed phase; however, intracolonic injection of lactate, but not other organic acids, recovered the rapid cell turnover comparable to that seen in untreated mice.

In this study, we attempted to apply this colonic response to starvation-refeeding to manipulate the colonic EC proliferation rate. We assumed that arrest of EC division decreased the damage from insults, and promotion of cell proliferation would induce efficient repair of injured tissue. In a colitis model induced by DSS in drinking water, intermittent fasting at the resolution phase increased the regeneration of ECs and decreased inflammation. At refeeding, delivery of lactate in the colon with feeding lactylated-high amylose further ameliorated the colitis. We also show evidence that lactate directly promotes EC proliferation and supports survival *in vitro*.

## Materials and Methods

### Mouse, induction of colitis, and intermittent fasting

All experiments were performed according to the Institutional Guidelines for the Care and Use of Laboratory Animals in Research with approval by the local ethics committee at the National Centre for Global Health and Medicine (NCGM, #16087). Male C57BL/6 mice were obtained from CLEA Japan Inc. (Tokyo, Japan) and fed normal bait (CE-2, CLEA Japan Inc.) *ad libitum* unless otherwise indicated. To induce acute colitis, mice in the experimental group were given 3.5% (w/v) DSS (Sigma-Aldrich Japan, Tokyo, Japan) in the drinking water for 5 days (starting on day 0) at 7 weeks of age. Fasting for 36 h was started by the removal of food from the cage at 8:00 PM on day 6, and the mice were refed at 8:00 AM on day 8. In preliminary experiments, fasting for 24 h was also examined but it gave unstable results compared with fasting for 36 h. During fasting, the mice were kept in plastic cages with bedding chips and drinking water. Colon samples were obtained on day 9. To avoid dietary fiber from refeeding food, a powder mixture of glucose and salt, based on oral rehydration solution formula, was used: 2.6 g sodium chloride, 2.9 g trisodium citrate dihydrate, 1.5 g potassium chloride, and 13.5 g anhydrous glucose. When this glucose mixture was given after starvation, mice regained weight at the similar level to those refed CE-2.^(15)^ Modified diets containing lactylated high-amylose maize starches (LHA) was prepared based on AIN93G with 15% (w/w) of high-amylose starch.^(17)^ For control of the lactylated diet, non-modified high-amylose maize starch (HA) was used. In enema experiments, DSS-treated mice were fasted for 36 h from day 6–8 and refed with glucose powder with rectal administration of organic acids or saline as a vehicle started at the timing of refeeding and repeated 5 times every 3 h on day 8. At rectal administration, mice were anesthetized with inhalation of sevoflurane (AbbVie Inc. Tokyo, Japan), and 0.1 ml of lactate (200 mM), acetate (600 mM), or butyrate (200 mM) was slowly administered via the rectum with a gavage tube.

### Histological analysis and evaluation of colitis

The whole colon was opened longitudinally, rolled from the distal end, and snap frozen. Frozen sections were prepared and stained with hematoxylin and eosin (H&E). To determine the number of crypts/area, at least 3 images from different fields were captured and the numbers of crypts or their cross sections were counted. The number of crypts was normalized with the mucosal area measured using ImageJ^®^ software (NIH). The mean of data obtained from one mouse was used as the representative number of crypts of each mouse. To measure total ulcer length, the length of mucosa without surface ECs was measured using ImageJ^®^, and summed.

### RT-PCR for cytokines

To measure the levels of cytokine transcripts, the rolled colon was snap frozen and subjected to total RNA extraction and quantitative reverse transcription polymerase chain reaction for IL-1β, IL-17, IL-6 and TNF-α. Total RNA was isolated with RNA-Bee RNA Isolation solvent (TEL-TEST, Inc., Friendswood, TX). After the RNA was treated with DNase I, double-stranded cDNA was synthesized using the High-Capacity cDNA Reverse Transcription Kit (Applied Biosystems, Foster City, CA). Expression levels were quantified with a TaqMan gene expression kit for TNF-α, IL-17, TGFβ1 and 18s rRNA (TaqMan Gene expression assay ID was NM_013693.2, Mm00439618_m1, Mm01178820_m1 and Hs99999901_s1, respectively) or a SYBR Green PCR Master Mix (for IL-6 and IL-1β) using an ABI Prism^®^ 7900HT Sequence Detection System. Primers used were as follows: mouse IL-6, 5'-TGGAGTCACAGAAGGAGTGGCT AAG and 5'-TCTGACCACAGTGAGGAATGTCCAC; and IL-1β, 5'-CAACCAACAAGTGATATTCTC and 5'-GATCCACAC TCTCCAGCTGCA. Threshold cycle numbers (Ct) were determined with Sequence Detector Software (ver. 1.7; Applied Biosystems) and transformed using the ΔCt/ΔΔCt method as described by the manufacturer, with 18s rRNA used as endogenous control.

### Three-dimensional primary culture of colon ECs

Colons were obtained from mice fed *ad libitum* and from mice after 36 h fasting. ECs were isolated from the colon and treated with 10 mM EDTA in Hank’s Balanced Salt Solution (Sigma Aldrich). Forty thousand cells/well in a 96-well half plate were cultured in 20% Matrigel (Corning, Tokyo, Japan) in a culture medium containing D-MEM/Media 199 (mixture of 4:1, Sigma and Lonza, Tokyo, Japan), 0.01 M HEPES, 1 × antibiotic-antimycotic (Gibco), 50 µg/ml gentamycin (Gibco) supplemented with 10% Hyclone Cosmic Calf serum (GE Healthcare), 50 ng/ml mEGF (Peprotech, Rocky Hill, NJ), 1 µg/ml hydrocortisone (Sigma-Aldrich), 2 µg/ml Transferrin (Sigma-Aldrich), and 5 nM sodium selenite (Sigma-Aldrich) in 5% CO_2_ and 5% O_2_ atmosphere. To study the effects of organic acids, lactate, butyrate, or acetate (10 mM at final concentration, pH was adjusted to 7 with NaOH, Wako, Japan) was added to the culture medium. Fresh medium (0.05 ml) with/without organic acids was added to the culture every 4 days (day 4, 8 and 12). After day 4 of culture, the number of colonies containing more than 10 living cells/well was counted everyday with a inverted microscope (IX71, Olympus, Tokyo, Japan). Cultures were performed in triplicate and data were presented as the mean ± SD, unless otherwise indicated in the legend.

### Western blotting

Lysis buffer containing 50 mM HEPES, 50 mM NaCl, 50 mM NaF, 10 mM Na_4_P_2_O_2_, 1 mM Na_3_VO_4_, and 5 mM EDTA, adjusted to pH 7.4, filter-sterilized, with 0.1% Triton X-100 and Complete Protease inhibitor (Roche Diagnostics, Tokyo, Japan) was used for protein extraction. Tissue samples were homogenized with 0.3 ml of lysis buffer in microtubes using a disposable pestle, centrifuged at 20,000 × *g* for 5 min, and the supernatant was used. After protein assay (Pearce protein assay kit, Thermo Fisher), protein concentration was adjusted to 2 mg/ml. Forty micrograms/lane was applied on a 10% precast gel (NuPAGE^®^ Novex gel, Thermo Fisher), and electrophoresis was performed with MOPS-SDS running buffer and transferred onto polyvinylidene difluoride (PVDF) membrane with a blotting apparatus HorizeBLOT (ATTO, Tokyo, Japan). Blotted membrane was blocked with PVDF blocking reagent (TOYOBO) for 60 min RT, staining was performed with anti-NDRG3 antibody (1:1,000, Abcam) and secondary goat anti-rabbit immunoglobulin G-horseradish peroxidase (IgG-HRP) (Santa Cruz) and HRP chemiluminescent reagent kit. For control, tubulin was visualized with anti-tubulin (1:1,000 Abcam #SC-2005) followed by goat anti-mouse IgG-HRP. Staining image was obtained using LAS3000 (Fujifilm, Tokyo, Japan). Semi-quantification from the images was performed using ImageJ (NIH) software.

### Statistical analysis

Statistical analyses were performed using GraphPad Prism 4 software (GraphPad Software, Inc) as indicated in the legends. Results were determined as significant when *p* values were less than 0.05.

## Results

### Intermittent fasting in resolution phase restored crypt regeneration and decreased cytokine production in colitis

**Fig. 1:**
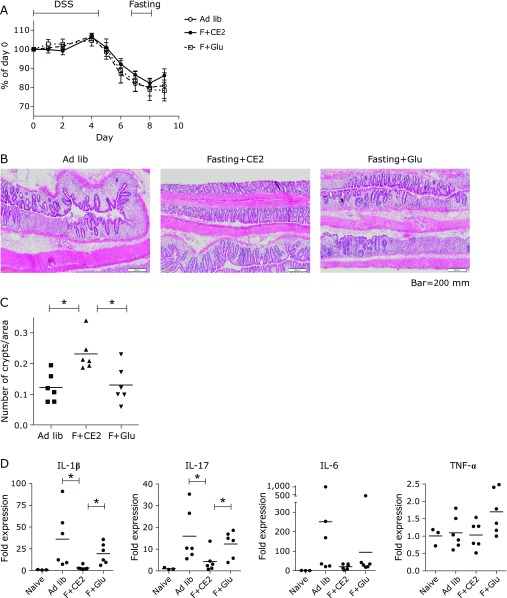
Intermittent fasting restored crypts and decreased cytokine production in colitis. (A) Body weight changes. Mice were fed CE-2 *ad libitum* without intermittent fasting (Ad lib); mice were fed CE-2, fasted at the indicated period and refed CE-2 (F + CE2); mice were fed CE-2, fasted and fed glucose and salt (F + Glu). Data are shown as mean ± SD of 6 mice for each group. A statistically significant difference the between F + CE2 and F + Glu groups (*p*<0.01) was seen on day 9 (two-way ANOVA with Bonferroni multiple comparisons). (B) Typical histological findings of the colon on day 9. Frozen sections were stained with hematoxylin & eosin. (C) Numbers of crypts were determined from the images as panel (B). Each dot represents an individual mouse and the short bar indicates the mean value (*p<0.02, Mann–Whitney *U* test). (D) Levels of cytokine transcripts in the colonic tissue on day 9. Data are shown as relative expression to naïve mice without induction of colitis. Each dot represents an individual mouse, and the short bar indicates the mean value. (*p<0.02, Mann–Whitney test). DSS, dextran sulfate sodium; IL, interleukin; TNF, tumor necrosis factor.

Treatment with DSS in drinking water induced severe body weight loss in all experimental groups. Clinical features including severity of diarrhea was similar in all groups. On day 9, mice refed with CE-2 showed a trend to gain more body weight than other groups (ad lib or refed glucose), and the difference from glucose-refed mice reached statistical significance (Fig. 1A). Histologically, ulceration with crypt loss was the most characteristic feature of this colitis model, and the fasting group (refed CE-2) showed higher numbers of crypts on day 9 (Fig. 1B and C). Glucose refeeding did not prevent severe crypt loss (Fig. 1B and C). When expression of inflammatory cytokines was evaluated at mRNA levels, expression of IL-1β and IL-17 was significantly increased in colitic tissues of the *ad libitum*-fed group (Fig. 1D). IL-6 also showed trend of increase in colitis, while TNF-α levels did not change. The fasting group showed dramatically decreased levels of IL-1β and IL-17. The glucose group showed lower levels of these cytokines compared with the *ad libitum*-fed group but this did not reach statistical significance. The differences in IL-1β and IL-17 between the fasting and glucose groups was statistically significant. In a previous study, we showed that mice refed with glucose were able to regain the weight but failed to show colonic EC hyperproliferation due to the lack of dietary fiber as substrate for production of lactate by lactic bacteria.^(18)^ Therefore, these results indicated that to suppress the crypt loss and inflammatory cytokines, only fasting plus nutritional signal was not enough, but the presence of microbiota-metabolizing fiber is required. Based on our previous result, we thought that lactate, as a metabolite of microbiota, would most likely promotes these anti-inflammatory effects.

### Delivery of lactate into the colon ameliorated colitis

**Fig. 2:**
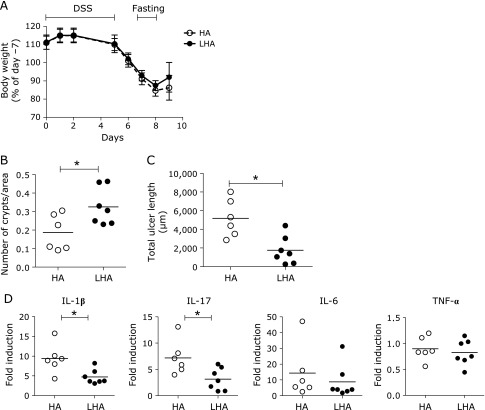
Delivery of lactate into the colon ameliorated colitis. (A) Body weight changes. Mice were fed CE-2, fasted at the indicated period and refed lactylated high-amylose maize starches (LHA, *n* = 7) or control high-amylose maize starches (HA, *n* = 6). Data are shown as mean ± SD. There was no statistically significant difference (two-way ANOVA). (B) Numbers of crypts were determined on day 9 from the hematoxylin & eosin (H&E)-stained frozen sections. Each dot represents an individual mouse, and the short bar indicates the mean value. *p<0.05 (Mann–Whitney *U* test). (C) Total ulcer length determined on day 9 from the H&E stained frozen sections prepared from the rolled whole colon. Each dot represents an individual mouse, and the short bar indicates the mean value. *p<0.02 (Mann–Whitney test). (D) Levels of cytokine transcripts in the colonic tissue on day 9. Data are shown as relative expression to naïve mice without induction of colitis. Each dot represents an individual mouse, and the short bar indicates the mean value. (*p<0.05, Mann–Whitney test). DSS, dextran sulfate sodium; IL, interleukin; TNF, tumor necrosis factor.

Next, we investigated whether increase in intracolonic lactate is beneficial to reduce colitis. For this purpose, mice were refed with modified diets containing LHA maize starches. High amylose is metabolized by microbiota in the colon and as a result, lactate is released in the colon. When compared with the control group, the lactate group showed no difference in changes in body weight (Fig. 2A); however, the number of crypts increased and total ulcer length decreased significantly (Fig. 2B and C). Levels of IL-1β and IL-17 were further decreased in the lactate group compared with the control group (Fig. 2D). These results suggest that fasting followed by the high concentration of lactate in the colon induced a beneficial effect in the resolution phase of DSS colitis. Since both control and lactate groups were given the same diet (CE-2) until fasting timing, which was the peak of colitis and body weight loss, this result also indicated that the action of lactate was not inhibition of EC damage but facilitation of crypt regeneration. To investigate the mechanism of lactate-induced resolution of colitis, we measured the expression of TGF-β, which was reported to play important role in the epithelial restitution and wound healing in inflammatory bowel disease.^(19)^ However, we did not find difference between lactate group and control group; Mean ± SD of relative expression of lactate group and control group were 1.17 ± 0.47 (*n* = 6) and 1.00 ± 0.59 (*n* = 7), respectively. Of interest, mice fed *ad libitum* had aggravated colitis (Fig. 1B), and these mice showed higher levels of TGF-β than refed groups (3.08 ± 0.65, *n* = 6, *p*<0.01, Mann–Whitney *U* test). This result suggested that beneficial effect of fasting or intraluminal lactate was not mediated by expression of TGF-β.

### Enema of lactate and butyrate increased the numbers of crypts in the colitis

**Fig. 3:**
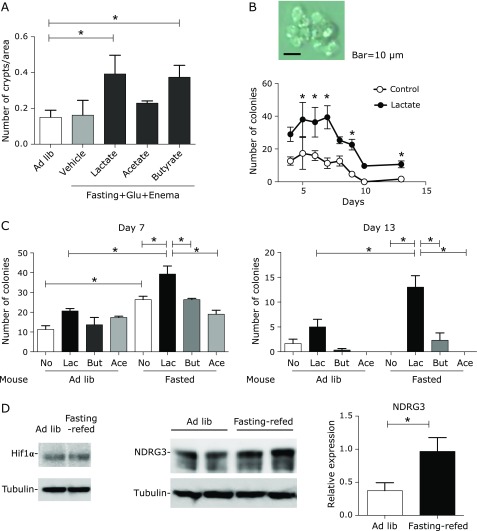
Direct effect of lactate on epithelial cells *in vivo* and *in vitro*. (A) DSS-treated mice were fasted for 36 h from day 6–8 and refed with glucose (Glu) powder with enema of organic acids or saline as a vehicle. Enema was started at the timing of refeeding and repeated every 3 h 5 times. Numbers of crypts were determined as in Fig. 1C. Data are shown as mean ± SD of 3 mice for each experimental condition. *p<0.05 (One way ANOVA with Tukey’s multiple comparison test). (B) Colonocytes from fasted (36 h) mice were cultured with or without lactate, and from day 4, number of colonies/well was counted every day. A typical colony is shown in the upper panel. Data are shown as mean ± SD of triplicated culture for each experimental condition. *p<0.05 (Two-way RM ANOVA with Bonferroni multiple comparisons). (C) Colonocytes from mice fed *ad libitum* (ad lib) or fasted for 36 h were cultured with Lactate (Lac), butyrate (But), acetate (Ace), or vehicle (No). Number of colonies/well on day 7 (left) and 13 (right) are shown. Data are shown as mean ± SD of triplicated culture for each experimental condition. *p<0.05 (one-way ANOVA with Tukey’s multiple comparison test). (D) Western blotting of the total colon homogenate from mice on day 9, with intermittent fasting (fasting-refed) or without (ad lib) for detection of Hif1α and NDRG3. Relative expression levels of NDRG3 to tubulin were quantified using samples from 4 mice for each condition and summarized in the graph (mean ± SD, *p<0.05, Mann–Whitney *U* test).

To confirm the direct effect of lactate in DSS colitis with fasting, an enema of lactate, acetate, or butyrate was performed in the mice refed with glucose. Intrarectal administration of lactate or butyrate, but not acetate, induced crypt regeneration to the same extent (Fig. 3A). The effect of butyrate matches previous reports that butyrate enema reduced severity of DSS colitis.^(20,21)^

### Lactate but not butyrate supports growth and survival of colonocytes

To further investigate the direct effect of lactate/butyrate on EC proliferation, we performed *in vitro* culture of colonocytes. For this purpose, a cell culture system representing normal colonocytes was required. Initially, we used cancer cell lines and organoid culture^(22)^ for *in vitro* experimental models of colonic ECs; however, it turned out to be difficult to detect the effect of organic acids in these systems because of strong autonomous growth signal in cancer cells and full growth factor supplementation to maintain the organoid culture. Therefore, we established a three-dimensional colonocyte culture system in hypoxic conditions without addition of supplements such as R-spondin, Wnt3A, or Noggin that are typically used in three-dimensional organic culture. In this system, ECs did not form spheres but grew as colonies by day 4 to day 7. These colonies were maintained for 3–4 days and eventually all cells died by day 13 (Fig. 3B). Thus, the numbers of cell aggregates at days 4–5 reflect the number of cells that have the potential to proliferate in the crypt, and numbers on days 8–10 reflect cell survival potential. Addition of lactate in this culture system increased the number of colonies and also prevented cell death on day 13 (Fig. 3C). This effect was not seen in the presence of butyrate or acetate. Of interest, increase in cell colonies by lactate was evident when colonocytes were prepared from fasting mice, but not *ad libitum*-fed mice. Thus, lactate has distinct action from butyrate in that it supports EC growth and survival after fasting.

### Lactate-sensing protein NDRG3 increases in the colon of fasted mice

Recently, a hypoxia-induced protein NDRG3 was reported to bind intracellular lactate. The abundance of NDRG3 correlated with the production of lactate, which resulted from the HIf1α–transcriptionally induced changes in metabolism, enhancing tumor cell growth and angiogenesis.^(23)^ We found that NDRG3 levels were elevated in the colitic mucosa of fasted-refed mice compared with mucosa in non-fasted mice (Fig. 3D). These data suggested that NDRG3 in the colon was increased in the presence of luminal lactate, mimicking the condition of hypoxia.

## Discussion

Recent studies indicated that dynamic metabolic processes regulate immune responses.^(24)^ Fasting-refeeding promotes the most drastic but universal changes of metabolism. In this study, we found that intermittent fasting in the resolution phase ameliorated the acute colitis. Herein, we discuss its possible mechanism of action including immune regulation by metabolic changes as well as the role of intracolonic organic acids in EC regeneration.

In our study, induction of colitis did not upregulate TNF-α, which indicated that transcription of TNF-α, a major target of NF-κB activation, was not significantly involved in this colitis model. On the other hand, induction of colitis evidently upregulated IL-1β, a hallmark cytokine of inflammasome activation, and it was suppressed in fasted mice, together with IL-17. IL-1β is well known to induce IL-17 critically.^(25)^ IL-6 is also a known downstream target of IL-1β, and changes in IL-6 generally showed similar pattern as IL-1β, although it did not reached statistically significant level (Fig. 1D). These results strongly suggest that this colitis model is mediated by inflammasome pathway, as previously reported.^(26)^ Further, ketone bodies, which are produced during states of energy deficit like fasting, are reported to block inflammasome-mediated inflammation,^(27)^ and Inflammasome is less activated in fasting humans.^(28)^ In summary, we speculate that activation of inflammasome, but not NF-κB, is the major event of this colitis model, and also a target of fasting therapy. During fasting, various metabolic changes take place. For example, phosphorylation of mTOR decreased after intermittent fasting^(1,29)^ and, simultaneously, AMPK is activated by fasting.^(1)^ Studies showed that mTOR facilitated adaptive immune response in CD4+ or CD8+ T cells, such as generation of Th1 and Th17 cells as well as Th2 cells.^(30)^ Further, inhibition of mTOR promotes Foxp3+ regulatory T cells.^(30)^ A recent study indicated that general controlled non-repressed (GCN2) kinase sensed acute amino acid starvation and plays roles in suppressing DSS colitis via induction of autophagy and inhibition of inflammasome activation.^(31)^ All these studies strongly suggest that intermittent fasting induced inhibition of mTOR and activation of AMPK and GCN2, with resulting decrease in production of IL-1β from activated inflammasome and reduced generation of Th17 cells. Although we did not assess the numbers of Treg cells, they might be involved in the amelioration of colitis by intermittent fasting. In addition, cycle of fasting is reported to increases hematopoietic and mesenchymal stem and progenitor cells, which are likely to contribute to the regeneration of various cell types/systems.^(7)^ Considering that mesenchymal stem cells have therapeutic effect in colitis models,^(32,33)^ effect of fasting on bone marrow cell may participate in the mechanism for better recovery in fasting mice.

On the other hand, in our study, refeeding of glucose mixture after intermittent fasting did not show a significant effect on the recovery from colitis. This indicated that not only fasting but also appropriate refeeding was important to exhibit anti-inflammatory effect on the colitis. Our previous study indicated that transient enhanced proliferation of colonocytes was induced by refeeding a high dietary fiber bait, but not glucose, and it was dependent on the lactate produced by fermentation of dietary fiber by *L. murinus*. Thus, in addition to the suppression of inflammation by the reduced nutrition/amino acid signals, it is strongly suggested that lactate promoted regeneration and supported survival of EC, as evidenced in the enema experiment *in vivo* and *in vitro* colony assay (Fig. 3). It also indicated the importance of the microbiota and its fermentation products in the rapid recovery from acute colitis with intermittent fasting. Among fermentation products, butyrate is already known to be a potential energy source of colonocytes and also has anti-inflammatory effect on local immune competent cells, such as Treg cells;^(34)^ the beneficial effect of butyrate enema was shown in patients with ulcerative colitis^(35)^ and DSS colitis models^(20,21)^ via its activity of epigenetic modification.^(36)^ Matching these reports, we also found that butyrate enema promoted cell proliferation to a level comparable to that induced by lactate. However, when we tested this in starvation-refed mice in a colitis-free condition, butyrate did not promote colonocyte proliferation at refeeding, while lactate did.^(15)^ In an *in vitro* colony formation assay performed with colonocytes from naïve mice, butyrate did not show a significant effect on cell growth and survival, which was clear in the lactate-treated cultures. These results indicated that butyrate-induced promotion of colonocyte proliferation might be evident in the presence of inflammation. On the other hand, lactate was able to promote EC proliferation and also cell survival without inflammation. Interestingly, lactate was most effective in colonocytes derived from fasted mice, as shown in Fig. 3. We assume that fasting modulated the cell condition to actively respond to lactate. To produce lactate in the colon, it was important to reduce the microbiota in the colon by fasting, where lactobacillus, which resides in the small intestine in *ad libitum*-fed mice, became able to grow transiently. In addition to this condition of microbiota to increase lactate, fasting may have some priming effect to utilize lactate at refeeding. Expression of NDRG3 may be one possible mechanism for this sort of priming. Binding of NDRG3 to lactate is likely to induce cell growth and have an anti-apoptotic effect. Since we could not detect upregulation of Hif1α signaling in the course of the colitis starvation-refed group, regulation of *NDRG3* gene expression in the colon needs further investigation.

The intermittent fasting in mice with DSS colitis was planned to be in agreement with the course of colitis, in which inflammation peaks on day 6–7 and ECs recover on day 8–9. As a result, reduced nutritional signals by fasting suppress inflammation when the colitis flares, while refeeding prompted tissue regeneration, which requires protein synthesis. Although clinical application of intermittent fasting is required to ensure its safety, our results have opened opportunities for a new therapeutic intervention for inflammatory disease.
